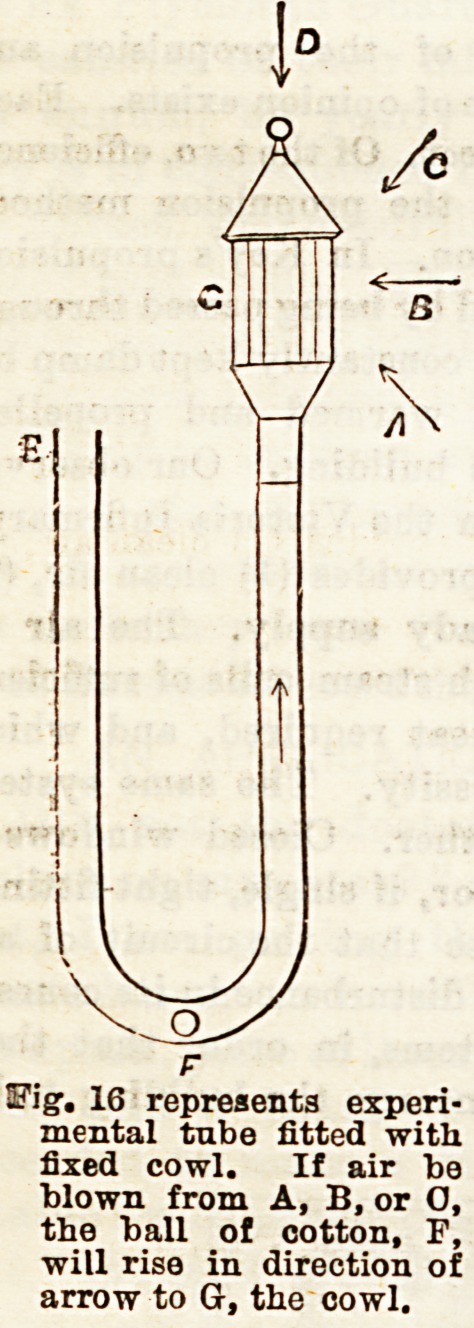# "The Hospital" Nursing Mirror

**Published:** 1896-05-23

**Authors:** 


### "The Hospital" Nursing Mirror.

Being the Extra Nursing Supplement of "The Hospital" Newspaper.

[Contributions for this Supplement should be addressed to the Editor, The Hospital, 428, Strand, London, W.C., and should have the word "Nursing" plainly written in left-hand top corner of the envelope.]

### News from the Nursing World.

### NURSES' HOME AT BRIGHTON.

The new wing of the Alexandra Hospital for Children at Brighton, of which the Duke and Duchess of Fife laid the first stone last week, will lie to the north of the main building, connected with it by a covered way; it will be of red brick, like the hospital, with timbered gables. The extra accommodation is badly wanted, and will add much to the comfort of the nursing staff. The home will contain twelve bed-rooms, with sitting-rooms and bath-rooms, and the cost is estimated at £1,500, including furnishing. Of this sum between £1,100 and £1,200 has already been collected. The Duke of Fife, on the day of the ceremony, paid a kindly compliment to the nursing profession, remarking that it was impossible to exaggerate the admiration felt by the Duchess and himself for those who devote their lives to nursing the sick. "Their patience, their self-denial, their devotion to duty, are quite beyond my power to praise, and it gives my wife and myself great pleasure to assist in any work that tends to make their daily lives easier and more comfortable."

### WOMEN'S WORK CONGRESS AT BERLIN.

At the forthcoming International Congress of Women's Work, to be held in Berlin in September of this year, on the third day the subject of the "Training ing of Medical Women, Dentists, Apothecaries, Sick Nurses, Midwives" will come under discussion, and model hospitals, ambulance appliances, &c., will be inspected. The programme for the whole week, from September 19th to 26th, is full of interesting matter, comprising as it does nearly every subject specially affecting women and their work and well-being.

### A PROTRACTED STRUGGLE.

The Longford Board of Guardians have been fighting the Irish Local Government Board for months past on the subject of the appointment of a trained nurse. The Local Government Board has insisted upon such an appointment being made, and at every board meeting the question comes up for discussion, is the cause of much warm language and excitement, is finally adjourned till another day, and the matter usually ends in the Local Government Board being requested to "reconsider their decision." Major Fair, the Local Government Board inspector, was present at the last meeting of the Board, and endeavoured to induce the guardians to advertise at once for a trained nurse at £30 per annum (at present the night nursing is done by an untrained girl, "absolutely incompetent"). One of the guardians remarked that the Local Government Board might "force a trained nurse upon us, but it would be an act of cruelty, and we should protest against it." In the end a resolution was again passed asking for a reconsideration of the matter. The "reconsideration" of the Local Government Board in these cases is generally very rightly the dissolving of the Board. That much reform is needed in the Longford Workhouse may be inferred from Major Fair's remark that the lunatic cells are "a shame and a disgrace."

### NURSING LECTURES IN THE PROVINCES.

"Lectures" are very popular just now, and every village aspires to have its "course" on "first aid" or "nursing." The classes are largely attended as a rule, and arouse much interest. There are two points of view from which to look at this thirst for knowledge. It is undoubtedly a good sign that people should wish to gain information on matters which are of very vital importance in daily life, and a better knowledge on hygiene and sanitary matters, and of "what to do till the doctor comes" is needed in most households; on the other hand, there is a good deal of truth in the old saying that a "little knowledge is a dangerous thing," and when the little knowledge is imparted by incompetent lecturers, as not infrequently happens, the danger becomes a real one indeed. The public need teaching, and are eager to learn, but a great responsibility rests on the instructors, and in many instances it is to be feared that they are by no means fitted for the work. There is not a word to be said against the wide diffusing of knowledge of the right kind, but those who are responsible for organising "lectures on nursing" should be very careful to choose as a lecturer one who, by full training and large experience, knows well the sort of information that should be given and the proper way to give it.

### CANNOCK.

At the last meeting of the Cannock Board of Guardians a letter was read from the Local Government Board calling attention to the insufficiency of the workhouse nursing staff and requesting the Board to appoint another nurse. The chairman, the Rev. E. J. Wrottesley (why have the customs of bygone days so great a charm for the clergy?), complained that the guardians were "being bullied by the Local Government Board inspector," the latter's chief point of contention being "that the patients were attended to by pauper inmates." Another clergyman guardian considered that "they were quite as capable of judging what should be done as was the inspector." "No action," it is reported, "was taken with regard to the letter." Is the Local Government Board content to have its recommendations treated with such scant attention?

### MIDDLESEX HOSPITAL CONVALESCENT HOME.

A Ladies' Committee has been formed, under the presidency of the Countess of Lathom, to issue purses to ladies and children willing to collect five guineas and upwards for presentation to H.R.H. the Duchess of York at the fête to be held at the Middlesex Hospital on July 1st, in aid of the Seaside Convalescent Home, which will be opened on that date.

### AN ENDOWED WARD FOR SICK NURSES.

The lady superintendent of the training school for nurses in connection with the Post Graduate Medical School and Hospital, New York, is issuing an appeal to the public for funds to endow a "nurses' ward" in the hospital in perpetuity, the beds to be free to any nurse holding a diploma from any American training school. Mrs. Van Zandt comments on the fact that in all New York there is no bed endowed for the use and benefit of trained nurses needing medical or surgical care. In England, too, sick nurses have not come in for as much care as might be expected in this respect. Very few hospitals boast even a properly-equipped special ward or sick-room for members of the staff, and the rank and file of the profession, when in time of poverty and illness have to take their chance with other applicants for admission. It would be a happy idea to carry out such a plan as that suggested by Mrs. Van Zandt at one of the London hospitals.

### INCREASE OF SALARY.

The Plymouth Guardians have decided to increase the salaries of their infirmary nurses from £23 to £30 per annum, by yearly instalments of £2 10s. This step is in consequence of the constant resignation of the nurses, who naturally have been anxious to obtain better-paid posts. The guardians will now be able to secure thoroughly competent women; but the trouble and expense incurred by a perpetual change of staff is not likely to be done away with in the future unless there is also an increase in the number of nurses. One of the guardians stated at the last meeting that the infirmary was "under-staffed," having "only three nurses where other places had ten."

### AN APPLICATION OF A POULTICE.

"Did not the doctor say I could have a linseed poultice on my chest?" asked the sick woman. "Yes, aunt, I am getting things ready." And the girl repeated over to herself, "Two basins, a spatula or iron spoon, piece of linen, linseed meal, kettle of boiling water, and a board—I think that's all." Then she proceeded to make a diminutive, neat little poultice suitable for the chest of a good-sized doll. "Here it is aunt," she said proudly; "I'm sure it's quite as good as the one the ambulance lecturer made." "Isn't it rather small?" said the sick woman. "My doctor said 'a good-sized one,' and what are you going to cover it in with?" "The doctor who taught us didn't say anything about applying it to the patient, aunt, and I'm sure I've got together everything he mentioned," and the successful pupil looked complacently at the cooling poultice. The aunt glanced at her niece. "Never mind, my dear, perhaps if you go through a second full course of lectures you'll be told how to make your poultices into useful applications; in the meantime I'll try one of my old cook's compositions, and (if you won't be offended) I think you would benefit by watching her put it on."

### A HOSPITAL FOR ALDERSHOT.

The foundation stone of a hospital for Aldershot is to be laid in July by the Duchess of Connaught. At present Aldershot, whose inhabitants number 17,000, has no hospital accommodation, even for cases of accident or emergency, and unfortunate sufferers have to undertake a railway journey if they require hospital treatment. The new building is estimated to cost £3,000, for which an appeal to the public is now being made. After the ceremony of laying the stone the Duchess has consented to receive purses towards the building fund.

### A HEALTHY SEASON.

A correspondent, who is a private nurse in La Plata, reports a year exceptionally free from sickness in that part of the world. There has been no cholera as last year, and, so far, very few cases of typhoid and dysentery. This unusually clean bill of health is attributed to a wet summer. Now the winter, always the more healthy season, is coming on, so private nurses are evidently at a discount for the present in the Argentine Republic.

### SHORT ITEMS.

The question of admitting a qualified medical woman as a member of the Pathological Society of London came up before the annual meeting on May 16th, and was decided in the negative by 43 votes to 21.—A new wing to the West Kirby Convalescent Home was opened on April 30th by Mr. T. B. Boyden. —The Queen has appointed the Princess Beatrice to the Governorship of the Isle of Wight.—A very successful ball was given on April 27th at the Royal Institute of Painters in Water Colours in aid of the Italian Hospital.—The Marlowe Dramatic Club gave a performance of Dion Boucicault's well-known Irish drama, "The Shaughraun," at St. George's Hall, Langham Place, on May 16th, in aid of the funds of the Great Northern Central Hospital. The hall was well filled by a very appreciative audience.—The Nottingham Board of Guardians are intending to contribute an annual subscription of £60 to the funds of the Nottingham and Notts District Nursing Association, the Local Government Board having fully sanctioned the proposal.—The sum of £39 12s. 3d., the result of a recent concert, has been handed over by the Sheffield Ladies' Choral Society to the funds of the Sheffield Nurses' Home and Training Institution, Glossop Road.—The Earl of Warwick presided over the annual meeting of the Leamington District Nursing Association. Lady Warwick takes much interest in the association, and herself contributes £50 a-year in order to maintain a second nurse for district work.—Miss Taylor, who has been for five years past nurse to the Honiton and District Rural Nursing Association, has given up her work and gone to Crediton. On her departure she was presented with a purse containing £39 10s. in token of the esteem and regard of those among whom she worked.—The foundation stones of a new general hospital were laid at Kettering the other day by the Duchess of Buccleuch and Lady Brenner. The site has been presented by the Duke of Buccleuch, and the money required (£10,000) is to be raised by public subscription. Lady Brenner has contributed £500 to the fund.—The late Lady Emily Williams has left by her will £50 to the Cheyne Hospital for Incurable Children, and the same sum to the Dorset County Hospital, while £25 each are bequeathed to the Devonshire Hospital, Buxton, and the Sussex County Hospital.—Miss Florence Nightingale was seventy-six on May 15th.—A monster petition, praying for the extension of the suffrage to women, has been on exhibition in Westminster Hall, signed by 257,000 women.

### Hygiene: For Nurses.

By John Glaister, M.D., F.F.P.S.G., D.P.H.Camb., Professor of Forensic Medicine and Public Health, St. Mungo's College, Glasgow, &c.

### VII.—VENTILATION — MODES APPLICABLE TO LIVING ROOMS, LARGE BUILDINGS, AND HOSPITALS.

In addition to means of ventilation applicable to ordinary window fittings, a nurse ought to be acquainted with special modes, so that she may intelligently know how to use them, when present. Of the special forms of ventilators, Sheringham's valve, Hatton's screen valve, and Tobin's tube deserve some notice.

Fig. 11.—Sheringham's Valve.

They are inserted into the outer wall of a room, so that one end communicates with the outer air and the other with the apartment. In the two former, the admission of air may be regulated by closing or opening the valvular opening, and in the latter, by a screen in the throat of the tube. Ventilation is also effected into the chimney by valve openings which open into it close to the ceiling of the room. Arnott's, Boyle's, and Buchan's valves are some of these. The valves, made of mica, or silk, are so laid that they only permit of the air passing from room to chimney. These act as extractors of foul air, the former as inlets of pure air.

Large Buildings and Hospitals.—Until recent years most of the large buildings and the older hospitals of this and other countries were singularly defective in means of ventilation. Nowadays ventilation is rightly put in the forefront of the constructor's design. It may be said of any large scheme of ventilation that none is in the least likely to be successful which does not unite the problems of heating and ventilation. This combination enables larger quantities of air to enter a building in the same time, and with greater safety, since the air is warmed. In towns and cities it is essential that such air be "screened" of its sooty particles. Hence, to effect this, it is necessary to employ mechanical aids to ventilation—so-called artificial ventilation—whether it be by revolving fans, jets of water, or the expanding force of compressed air.

Fig. 12.—Hatton's "Screen" Valve.

Mechanically-aided ventilation schemes are (1) those where fresh air, washed and warmed, or warmed without washing, is propelled into buildings and the foul air driven out by special channels; or (2) those in which the foul air is extracted, the fresh air entering to fill its place. In the former the motive power acts at the beginning of the air circuit, and in the latter at the end; and in the former that motive power must be some form of mechanical force, whereas in the latter it may be mechanical force—as a fan—or the extracting power of a fire or furnace. The following diagrammatic figure will assist the student:—

Fig. 13.—Tobin's Tube.

Fig. 14.—A. The air-propelling and extracting forces are fans, acting in direction of arrows. B. The air-propelling force is a fan, the extracting force a furnace at base of tower. C. The air extracting force is a furnace, by which air is drawn through whole circuit; or it may be replaced by a revolving fan.

Regarding the respective merits of the propulsion and extraction methods, much difference of opinion exists. Each has merit, and each its probable defect. Of the two, efficiency would appear to be on the side of the propulsion method, because of the steadiness of its action. In Key's propulsion system the air is washed and screened by being passed through a screen composed of fibre, which is constantly kept damp by an automatic dripper; it is then warmed and propelled forward, free of "smuts," into the building. Our observations of this system as carried out in the Victoria Infirmary, Glasgow, enable us to say that it provides (1) clean air, (2) evenly warmed air, and (3) a steady supply. The air is warmed in passing over and through steam coils of sufficient radiating surface to give all the heat required, and which may be regulated according to necessity. The same system can provide cooled air in hot weather. Closed windows—double if necessary—and double, or, if single, tight-fitting, doors are requisite in this system, so that the circuit of air may be subjected to the minimum disturbance in its course. Indeed it may be said of both systems, in order that they should work efficiently, that the nearer the building to be

Fig. 15.—Galton's Stove.

ventilated conforms to the parallel of a tight-fitting box with suitable openings, the more thorough is the ventilation. Open windows and doors destroy, in great measure, the continuity of air-current, and are the prime causes of failure and inefficiency.

The source of air supply in populous places is always a matter of importance where the air is not washed and screened. Air is always more foul the nearer to the surface of the ground. For this reason the supply is sometimes obtained by high towers, as in the French House of Deputies and the Madison Theatre, Melbourne. The British House of Commons obtains its air-supply from, practically, the ground level. The advantage of washing the air is best seen in foggy weather. In the Victoria Infirmary the atmosphere is perfectly clear, although a dense fog may obtain outside.

Attempts have been made to adopt the plan of washing and warming the air in the ventilation of clubs and dwelling-houses. It cannot be said that they have been, as yet, successful. The plan usually adopted is to take the outside air from the ground level, pass it through revolving water-spirts situated in the walls of the building, then through and over hot water coils, until it reaches the apartment through a grated opening at the top of an artificial dado. Such a plan is exposed to the attendant risks of frost.

Means have also been devised to supply living rooms with warmed air by utilising the spent heat of the fire or stove. By means of a special duct either surrounding or in close proximity to the source of heat, the entering fresh air is warmed, and is passed into the room either at the ceiling-level or close to the fireplace. Of these, Galton's stove, George's Calorigen, Bond's Thermohydric stove, and the Canadian school stove may be taken as types. Fig. 15 represents Galton's stove in operation; the fresh warmed air, entering at the ceiling, is led in from the open by a special duct which surrounds the flue.

Fig. 16 represents experimental tube fitted with fixed cowl. If air be blown from A, B, or C, the ball of cotton, F, will rise in direction of arrow to G, the cowl.

Churches, schools, halls, and similar large buildings are often ventilated by roof-ridge ventilators, which are either fixed, or are movable by the wind. The movable ventilators either act in weather-cock fashion or in a rotatory manner. Much has been written for and against this plan of ventilation, but, in our opinion, where failure has had to be registered, that must be attributed to inadequacy in size of ventilators and accompanying ducts. All roof-ridge ventilators act by the perflating action of atmospheric air currents, which, in our climate, seldom move with a less velocity than 15 to 20 miles an hour. Action by perflation is illustrated by Fig. 5 of a former paper, and by Fig. 16. The main objections to this system are (1) irregularity of action, and (2) back-draughts. The latter, however, may be prevented by suitable valvular arrangements and periodic inspection of apparatus.

### Where to Go.

Guy's Hospital.—The Duchess of Albany will open a fancy fair in aid of the Re-endowment Fund for Guy's Hospital, at Shortlands, Kent, on May 29th.

Great Northern Central Hospital.—A grand morning concert will take place at the Queen's Hall on June 11th, in aid of the Ladies' Endowment Fund.

The Working Ladies' Guild.—The summer sale of the Working Ladies' Guild will be held at 7, St. James's Square, by the kind permission of Lord Egerton of Tatton and the Duchess of Buckingham, on June 2nd, 3rd, and 4th. Open from twelve to seven each day.

### Workhouse Infirmary Nursing Association.

### ANNUAL GATHERING.

The annual gathering of the Mary Adelaide Nurses, always a pleasant occasion, this year took place on May 15th, by kind of permission of Earl and Countess Brownlow, at 8, Carlton House Terrace. There were fewer nurses present than usual for various reasons, many being unable to leave their work. Lady Lothian acted as hostess in the absence of Lady Brownlow. At seven o'clock there was tea, after which the nurses much enjoyed seeing the house and a walk on the terrace, and at eight o'clock everyone assembled in the hall to witness the presentation of medals and gratuities to the nurses by Lady Lothian. Of the nineteen thus honoured all but one were trained by the association.

Miss Twining first addressed the nurses, referring especially to the pleasure all felt at seeing Lady Lothian again, who, though a foundress of the association, had been prevented by ill-health from taking any active part in the work for some years. Miss Twining read some interesting extracts from the nurses' letters, and alluded to the improvements affected at Newton Abbot since the introduction of trained nursing, Nurse Jeffery, the head nurse, being one of the recipients of the medal. An excellent report of her work had been received from the clerk to the guardians.

Dr. Savill spoke on the very important subject of the Superannuation Bill for Poor Law Officers now before Parliament, and pointed out that it did not provide for the interests of nurses proportionately with those of other officers included in its benefits. He urged the nurses to join the Royal National Pension Fund for Nurses, drawing a comparison between the advantages offered by this fund and the proposals of the Poor Law Superannuation Bill.

Finally, Lady Lothian addressed a few encouraging and kindly words to the nurses, reminding them of Agnes Jones, the great pioneer of workhouse nursing, and showing how noble and great might be their work of tending the sick and suffering poor.

A pleasant little entertainment followed. Mr. Guy Stephenson's comic songs were most amusing, and a clever whistling duet was warmly applauded. The duologue "A Joint Household" was given with spirit, and was much enjoyed. The Association nurses are generally on these occasions to be noted for their neat and nurse-like appearance; there is an absence among them of the fringes and fly-away caps which are too often to be seen where nurses gather together. The St. George's-in-the-East pink uniform was in force; one bright red uniform (a little startling on a close inspection) with a high stiff white cap and spotless apron looked picturesque amid the crowd. A good many visitors were present, amongst whom were Lady Wantage, the Hon. Mrs. J. G. Talbot, Lady Belhaven, Miss Twining, Dr. Savill, Miss Wilson, Miss Gill, Miss Rosalind Paget, Miss Brierly and Mrs. Nichol, Miss de Pledge, Miss Moir, Miss C. J. Wood, and Miss Wesley. Each nurse on leaving was presented by Lady Lothian with a bouquet of flowers, provided by Lady Brownlow.

### Appointments.

Llanelly General Hospital.—Miss Griffiths has been appointed Matron to this hospital. She has for some time held the post of head nurse at the Denbighshire Infirmary.

Enfield Cottage Hospital.—Miss E. F. Neve has been appointed Matron of the Enfield Cottage Hospital. Miss Neve received her training at the London Hospital, and has worked in connection with the same institution first on the private and then on the hospital staff until the present time. We wish Miss Neve all success in her new duties.

### Some Aspects of Life in a South African Asylum.

By One of the Attendants.

It may, perhaps, interest readers of The Hospital to know some of the details of life in a South African asylum, as compared with the routine of an English asylum, and to take an unprejudiced view of the advantages and disadvantages thereof, for emigration is becoming more common among nurses, even asylum nurses, than it used to be ; and it appears to me, from what I have myself observed, that nurses are apt to come out here with very incorrect notions of what they are coming to. They expect to find everything the same as it is at home—which is unreasonable—and when they discover that it is not so, in their annoyance at the things they miss, they are apt to overlook the things that they gain.

Within the last five years (which covers my personal experience) very great improvements have been made in South African asylums, and there is at the present time a new asylum in course of erection near Capetown, which is built, I believe, on the lines of the best English asylums, and will be second to none. In former days, however, any building that could be had was adapted to the purpose, and many unavoidable inconveniences were the result. The Grahamstown Asylum, for instance, had been a barracks, and was in many ways unsuited for an asylum. There were no proper quarters for matron and attendants, the kitchen and laundry were too small and too far away, there were no modern appliances in the shape of gas, hot-water pipes, &c. As a natural consequence, the actual manual labour of the nurses and attendants was doubled, and much of their time taken up with the invention of shifts and contrivances. To the colonial nurses this was not such a hardship, as they had never known the many little smoothnesses of life in an English institution; but to nurses fresh from the commodious asylums and hospitals at home these small discomforts and privations were real trials, and being unprepared for them made them even harder to bear.

As I have already said, such great improvements have been made, and are being daily made, that most of these discomforts and privations are things of the past; but still, comfort is not understood in the colony as it is at home; civilisation has not yet made luxuries indispensable, and, in any case, the conditions of life are bound to be different in a different country with a different climate. It would be well for all nurses intending to emigrate if they had these facts firmly fixed in their minds.

To begin with the climate. The heat in summer is very trying to some English people, and equally trying to many colonial people, including patients. It always seemed to me that patients were more excitable, and more difficult to control, in the very hottest weather, when one felt least able to cope with them. A struggle with a refractory patient when the temperature is 104 degrees in the shade, is anything but an agreeable exercise; and there is a strong temptation to let the refractory patient go her refractory way unrestrained. This is especially the case with the native patients, to whom heat is life. In the winter they cower in the corners, rolled up in their garments, as only native patients know how to roll themselves, quiet and amenable; but wait for the glaring days of summer. It is then that they dance and sing, and clap their hands, and waltz bareheaded in the broiling sun, and refuse to listen to the voice of the charmer, charm she never so wisely; and it is then that they continue their frolics right through the summer night, especially if a silver moonbeam finds its way into the dormitory.

Then the colony is subject to very terrible droughts, which involve much labour and privation. Water is scarce when most needed. It must be economised, it must be fetched long distances in buckets, and when it arrives it is hard, or brackish, or impure. Drought, in its true sense, is unknown in England.

Moreover, in intense heat all the routine of life becomes hard labour. The walks, the entertainments, the cricket matches, even the regular meals and ordinary housework, become more or less burdensome with the above-named temperature. On the other hand, however, there is the counterbalancing advantage of life in the open air. The asylums are provided with broad sheltered verandahs (here called "stoeps") opening on to the grass courts, and here the patients can spend their whole time when not employed. All the imbecile, helpless, useless patients can lie and sit on these stoeps, or in the grass court, the whole day long, and the nurses who have charge of them sit there also, while the doors and windows of the living rooms stand open to the outer air. Even in wet weather they can remain on the stoep if it is well roofed, and if one side of the building is cold and windy the other side will probably be sheltered. To my mind this open air life has advantages which make up for many disadvantages. Patients who cannot be taken out for country walks need never be shut up in rooms; the most refractory can have fresh air all day long, and can work off their excitement running up and down the stoeps or about the court, where they can do no damage if properly supervised.

To pass to another subject, it is true that the comfort of nurses and attendants was not studied in the colonial institutions of former days. A great deal more is done for them now, perhaps everything that could be done, in some institutions; but, as I have said before, even in private homes, comfort is not understood as we understand it in England, and English nurses will be likely to miss many little comforts and enjoyments to which they are accustomed. This will be especially the case when they are off duty, and have no places of entertainment to pass a few hours, no cheap trips for the day or the Sunday, no public gardens or good shops within an easy walk, and when they realize that all their old friends are 6,000 miles away, and that the new ones do not take their place.

To set against this, is the consideration—an important one for those who are laying up for the future or helping their families—that an asylum nurse in South Africa has twice the salary that she earns at the same work in England. The youngest nurse begins with £40 per annum, board, lodging, and washing of course included. A permanent night nurse has £50, and a charge nurse £60. After one year's service there is a rise of salary, and after three another. In this respect the colonial asylums are an example to the English.

In conclusion, I would say to anyone who thinks of coming out here to work in an institution of any kind, do not expect too much, and there is no need for disappointment. Life in any place is pretty much what we make it ourselves; and circumstances, like mountains, depend for their appearance entirely on the point of view from which we look at them. A mountain that is very steep on one side may be quite easy of ascent on the other.

There is no doubt that, in spite of the rapid strides of civilisation, life in the colony is rougher and more primitive than at home. Reason and common sense will show us that two generations of men cannot be expected to have done what fifty generations have hardly attained to, and to my mind there is something wholesome and invigorating in returning to a simpler and less conventional mode of life, and in wrestling hand to hand with the primal forces of nature. Even the unpleasant necessity of carrying a bucket of water half a mile, instead of merely turning a tap, may add an element of strength to our character, and may teach us the useful lesson that all the comforts and conveniences of modern civilisation were won for us by the patient striving of those who went before us.

### Trained Nurses' Clinic.

V.—THE NURSING OF BRONCHITIS—(*Continued*).

At the commencement of an acute attack of bronchitis every hour is of value, and much depends on the promptitude with which the doctor's orders are carried out. In hospitals the difficulties are reduced to a minimum by science, method, and skill, but in private and district nursing they often seem at first sight insurmountable. The trained nurse fresh from ward work is appalled to find every appliance on which she has hitherto relied, conspicuously absent from most private dwellings, and she has to contrive for herself such substitutes as can be most readily procured and utilised. She has also to face an amount of responsibility hitherto unknown to her, for instead of working within call of the doctor, and in close association with other competent women, she, for the first time in her career, stands alone. She has still to observe accurately the directions of the medical man, but she has, in addition to this, to anticipate his wishes, and to meet all emergencies promptly. It is impossible for those who have not experienced it to realise the change which instantaneously occurs in the position of a nurse who leaves the "ranks" in a first-class hospital school for the new sphere where so much more is expected of her. But the strangeness soon wears off, and lessons of self-reliance are quickly learnt by a woman who has already a foundation of knowledge and common sense.

Without this thorough preliminary training no one can conscientiously consider herself a fit and proper district or private nurse, but bronchitis is too common and general a complaint for its victims to be exclusively looked after by trained nurses, and it comes within the experience of most people.

One of the first duties of those nursing these acute cases consists in the immediate application of such remedies as are ordered, both internal and external. With regard to the latter it is of the first importance that they should be not only promptly put on but frequently renewed until all urgent symptoms have abated. Fomentations and poultices, &c., often give much relief and comfort, and their value depends entirely on the nurse's deftness in renewing them before the patient is conscious of their growing cold. This detail is apt to be overlooked by amateurs, who cherish an idea that it is wrong to remove the original poultice until it is quite chilled, an opinion somewhat detrimental to the sick person's comfort.

The temperature of the room needs incessant care, and the atmosphere should be kept as pure as possible, therefore an even temperature must be registered by the thermometer, whilst "closeness" is avoided. Special pains will be required in removing the dust from floor and furniture, otherwise the patient's abnormally sensitive bronchial mucous membrane will be needlessly irritated. The position of the bed is of material importance, but is a matter difficult of arrangement in very small rooms where the place it occupies is dependent on the exigencies of the floor space.

Even under these circumstances an ingenious nurse can sometimes improve matters by reversing the bedding and persuading the patient to have her pillows put at the foot of the bed if that end seems to have advantages over the other.

For the maintenance of the temperature of the room, for the punctual administration of food and medicine, it is essential that a person suffering from acute bronchitis should be well looked after at night, and arrangements to secure this care often devolves on the nurse. She should, without hesitation, give her employers an early intimation of the fact, and help them to manage so that whether it be a private house or cottage the case shall be adequately looked after during the night, more especially during the chill hours which precede the dawn.

Unfortunately patients are often unaware of the serious nature of some forms of bronchitis, and the doctor is only summoned when the disease has made considerable progress.

Healthy persons with no other malady often pull through severe attacks very quickly to the triumph of doctor and nurse, but for very young and very old people, as well as those previously affected with some other disease, the outlook is sufficiently serious. Good nursing is capable of vastly assisting the medical treatment of such cases as these, and is, in fact, absolutely essential.

With regard to the different degree in which the bronchi may be affected, and the varying extent of area over which the disease extends, most trained nurses are well informed. The nursing which comes within their special province is of such peculiar value in bronchitis that too much stress cannot be laid on the necessity for constant care from the onset to the conclusion of an attack. Slight cases should never be lightly thought of, but should be accorded such attention as will preclude the possibility of their growing more serious by reason of primary neglect.

### Mission to Deep Sea Fishermen.

The annual meeting of this society was held in Exeter Hall on Tuesday, Sir Joseph Pease taking the chair. The Duchess of Albany presented "awards" to the smacksmen, many of whom were on the platform. The accounts of the work done among the fisherfolk by Drs. Grenfell and Willway and Mr. Frank Wilson are always stirring to listen to. There is something about their graphic descriptions which goes home to the hearts of the hearers, and ought to result in arousing plenty of public interest. Active preparations are now being made for carrying on the summer work in Labrador, and the doctors return almost at once to reopen the hospitals and bring the two steamers, the *Sir Donald* and the *Princess May*, down from their winter quarters. The season has been a bad one, the seal fishery having been very poor, so that times are extra hard. There are many gifts in kind which would be most acceptable to the mission (whose headquarters are at Bridge House, 181, Queen Victoria Street, E.C.); amongst others, a good oil lantern and lantern slides are wanted for the entertainments with which the dreary winters are brightened for the dwellers in these lonely regions. Knitted garments, too, are ever acceptable, as well as books and magazines.

### Presentations.

A pleasant gathering took place on May 9th at the City of Dublin Nursing Institution, 27, Upper Baggot-street, when Mrs. Treacy, the lady superintendent, was presented by her nurses with an address, illuminated by Mrs. Butler, one of the staff, and also a pretty *tête-à-tête* tea service. Mr. Wheeler, one of the directors of the institution, presided, and the Right Hon. Lord Justice Gibbon, the Countess of Bantry, the Countess of Annesley, Lady Ardilaun, Florence Lady Power, and Miss Power were among the guests. The gifts were handed by Mrs. Butler to Mrs. Treacy, who, in thanking the nurses warmly for their kind thought, remarked that she and they being, as nurses, more separated from their families than many women, their thoughts and affections centred specially round the home; she believed they became more attached to each other with every day, and happier for being fully occupied in working for the good of others. She was proud to have the charge of so numerous a "family," and the kindly tokens that day given to her would be always reckoned amongst her chiefest treasures. After tea the nurses sang some glees and duets.

### Minor Appointments.

St. Saviour's Infirmary, East Dulwich Grove, S.E.—Miss A. E. Little has been appointed Night Superintendent at this infirmary. Miss Little was trained at St. Mary's Hospital, Paddington, and has since for two years worked as sister at the Paddington Infirmary.

### Everybody's Opinion.

[Correspondence on all subjects is invited, but we cannot in any way be responsible for the opinions expressed by our correspondents. No communications can be entertained if the name and address of the correspondent is not given, or unless one side of the paper only be written on.]

### THE DIFFICULTIES OF A PROVINCIAL MATRON.

A "CHARGE NURSE" writes: It is quite evident from the article on the above subject that the difficulties arise from the matron not understanding the limit of her powers. Having risen from the position of sister to that of matron, she adopts the rôle of mistress of the institution (which is right providing she goes about it in proper manner), putting everyone down as ignorant and knowing nothing; this is clearly seen by the manner in which she speaks of her nurses, who giggle, gossip, and work "like third-rate shop-girls with an occasional touch of the cheeky barmaid." This sentence casts a grave reflection upon the choice of nurses by her predecessor, showing that the "Provincial Matron" has not learnt that "Charity suffereth long and is kind." A sister taking the control of a "fine London ward," finds many things done that are not in accordance to her taste or wishes, and she has to work quietly and steadily on until she can arrange all things her own way. This applies also to a matron, only in a wider sense. If a ward requires regulation, how much more would a hospital? A newly-fledged matron intends to carry all before her, but by bitter experience she finds that success in a matron's career is only obtained by patient tact, quiet firmness, and strict justice. Hospital etiquette asks the matron to leave the ward when the "young house surgeon stalks in," so it is the doctor that is master of the situation, and not the "vulgar charge nurse." Let the "Provincial Matron" remember that "courtesy begets courtesy," and that if she treats her doctor as a gentleman, and her charge nurses and probationers as ladies, and not as "third-rate shop-girls," she will not find it such a great difficulty to obtain the tone and dicipline she would wish to prevail.

### ENGLISH CONVALESCENT HOMES.

"MARAH" writes: Can you spare a small corner in your popular paper for a few words on convalescent homes and the system of admission thereto? I propose to deal with the home at Eastbourne and the two Jewish homes at Norwood and Brighton respectively. All three I know, from personal visits and reliable information, to be excellent institutions where patients are kindly received and ably treated. Nevertheless a most important defect in the admission of patients is common to all three. The Eastbourne Home is, like our voluntary hospitals, undenominational. The authorities have wisely ordained that advanced cases of phthisis, as being infectious to others, shall not be admitted. But surely the consultants at Brompton are better judges of when phthisis reaches the infectious stage than any ordinary practitioners! No physician would send an infectious invalid to endanger the health of convalescents. For such cases the Ventnor and other special consumptive homes are available. The resources of Ventnor are limited, consequently physicians order Eastbourne, Brighton, or Hastings in the early stages of the complaint, certifying that the "spume" is not hurtful to others. Such a certificate is surely of far higher medical value than any opinion of an ordinary practitioner, who may be working in private cases under that very consultant's advice. Yet the rule for admission to Eastbourne, as also to the Jewish convalescent homes at Brighton and Norwood, is that no patient shall be admitted unless examined and passed by the doctor belonging to the home or examining depôt in London, who usually kindly performs this work gratuitously. Of course, this "convalescent home" doctor cannot have the same experience as the Brompton consultants, who send down their certificates with patients. It is the consultant who should question the ordinary practitioner's verdict, not the practitioner the consultant's. The governors of the homes either cannot or will not see this common-sense view, and consequently enforce a patient being overhauled and re-examined by the junior when he is fresh from the superior's treatment. The first bad result of this unwise rule is that one consultant at Brompton will no longer sign a certificate for a patient's admission to Eastbourne. His words are, "I will not waste my time in cavilling with an ordinary practitioner as to the stage of phthisis of my patients. If I write a certificate that my patient is non-infectious that should suffice; my authority is not to be questioned by any ordinary practitioner, hence, nothing shall induce me to sign another certificate for admission to the Eastbourne Convalescent Home." "Quite right, doctor, but how about the patient? You order Eastbourne air?" "I do." "Then get a few ladies and gentlemen to unite (if you cannot afford it alone) in taking private rooms for your *protégé*; I would rather help my patient that way than be bothered any more by useless correspondence"; and the lady took the physician's advice, withdrew her annual subscription from the Eastbourne Home, and sent her *protégé* elsewhere—the first of probably many secessions from the subscribers to a really fine institution. Bad as this system is, that prevalent at the Jewish convalescent homes is worse. These two branches at Brighton and Norwood are open gratuitously to all Jewish patients—men, women, or children. An able matron, both at Brighton and Norwood, really does her best to carry out any doctor's orders for those under her care, and always succeeds in making the convalescents happy. All this good work is spoilt by the scheme of admission, which necessitates every patient, even those recommended by a physician from a hospital or ordinary practitioner, journeying to Duke's Place Synagogue to be re-examined by an ordinary practitioner (appointed by the ruling council), which gentleman kindly gives his services gratuitously. If the committee will have this crucial test for the invalids they should employ a physician equal in knowledge and position to members of the hospital medical staffs. Of course, most of the applicants are of the poorest working classes, and as there are (especially in summer) many more applicants than beds, numbers have to trudge to Duke's Place, Aldgate, from all parts of London at great expense and trouble (not counting waste of time and strength) many times before the doctor in authority can sign the letter (subscriber's) for admission to the home. Thus many a promising case from the hospital falls back into a hopeless condition through this delay and worry. And why? Because the committee prefer to continue the old system sooner than take a little trouble to revise it or adopt new arrangements. I would suggest that admission should bear these lines: Anyone requiring admission to a convalescent home for a patient should write and notify such requisition (in early stage of illness) to the president of the committee, or to the home itself, stating nature of illness and asking probable date of vacancy. The committee should date and number applicants' letters as they do for Brompton Hospital. If full for a month, or more, the president could write "No room for six weeks," &c. Should it be imperative that the invalid go to the seaside or country before there be a vacancy at these homes, then the applicant must accept the inevitable. Nothing more is needed, no second order or examination; only a little trouble to the subscriber, and a small sum of money to give a patient the greatest boon in life—rest and quiet happiness after illness in pure, sweet air. If the patient can wait without harm till his numbered turn comes round it is easy enough. Nothing but a letter the evening before his arrival to the matron with details of case and his safe transport to the home. This is but a rude scheme of a young thinker and worker amongst the poor. I sincerely hope, Mr. Editor, some of your many clever and experienced readers will give their time and a little thought to the evil amongst us, and try to remedy it by a larger and better scheme that real friends of the poor may and can suggest.

[We insert the above, but it is clear that there is another side to the question which hospital authorities cannot afford to ignore. "Marah" clearly has a profound respect for the all-seeing wisdom and the unimpeachable integrity of the physicians to the Brompton Hospital, but it must be remembered that patients are sent to convalescent homes not only by practitioners of such exalted status, but by others who may fairly be called "ordinary practitioners," although we do not use that term in the same way as "Marah" does as expressing inferiority, and plain as may seem the distinction between these two classes of practitioners, it is one which in reality does not exist. No line can be drawn, and in such an important matter as the protection of convalescents from tuberculous infection, such as it is, each convalescent hospital must act for itself and through its own officers. It is perfectly well known that different medical men take the most widely different views as to the possible infectiveness of tuberculous patients, and many of them would think that they were doing very wrong if they deprived a sick man of the advantage of a visit to a convalescent home merely because of some "fanciful" notion about infection. The only way, then, by which an institution can ensure uniformity of practice in regard to the admission of tuberculous patients is by making all the cases be examined by their own officer. The reference to the Brompton Hospital in regard to this matter is peculiarly unhappy, for it is generally believed that the staff of that institution is riddled with scepticism in regard to the infectiousness of tuberculous patients.—Ed. *T.H.*]

### Notes and Queries.

The contents of the Editor's Letter-box have now reached such unwieldy proportions that it has become necessary to establish a hard and fast rule regarding Answers to Correspondents. In future, all questions requiring replies will continue to be answered in this column without any fee. If an answer is required by letter, a fee of half-a-crown must be enclosed with the note containing the enquiry. We are always pleased to help our numerous correspondents to the fullest extent, and we can trust them to sympathise in the overwhelming amount of writing which makes the new rules a necessity. Every communication must be accompanied by the writer's name and address, otherwise it will receive no attention.

### Queries.

(46) *Monthly Nursing.*—Where should I apply for a post as monthly nurse or midwife? I am fully qualified.—(*G. A. W.*)

(47) *Nursing Abroad.*—I am anxious to go abroad, and shall be very grateful if you will tell me how I ought to manage? I hold a three-years' certificate from a large general hospital.—*Hopeful Abroad.*

(48) *Daily Nursing.*—I am anxious to obtain daily or hourly nursing (not district) in a good London locality, and should be grateful for any information on the subject?—*Africa.*

(49) *Consumption.*—Will you tell me where I can obtain the "prepared paper" recommended in The Hospital some time since for the use of consumptive patients instead of pocket handkerchiefs?—*Birkdale.*

(50) *Com ination Furniture.*—Having read your article in The Hospital for September 28th, 1895, on "Furniture and Fittings for Nurses' Rooms," I should be glad if you would tell me what firm would undertake such fittings? I am wishing to furnish a bed-sitting-room in the manner described.—*Nurse.*

(51) *Orphanages.*—I want to get two fatherless boys into a home. What steps should I take?—*A. S.*

(52) *Monthly Nursing.*—I am anxious to become a certificated monthly nurse. Please tell me what to do.—*Nurse B.*

*Summer Holidays.*—*N.H.R.U.*—If Sister Florence will send her name and address, we shall be happy to publish her letter on this subject. Full name and address is required from correspondents, as "evidence of good faith," not for publication, if that be not wished.

*Midwifery Training.*—M.S., *Newcastle*, is reminded that name and address must accompany all communications for this column.

### Answers.

(46) *Monthly Nursing.* (*G. A. W.*)—Watch the advertisements in The Hospital "Mirror," or apply to the matrons of the various lying-in hospitals, of which you will find a list in "Burdett's Hospitals and Charities."

(47) *Nursing Abroad* (*Hopeful Abroad.*)—You had better advertise, and watch the advertisement columns. We are always warning nurses not to attempt to go abroad on the chance of obtaining private nursing. It would be most unwise unless you have private means. Read what has been said in back numbers of The Hospital "Nursing Supplement" over and over again on this subject. If you wish to become a Nursing Sister in the army or navy, write in the former case to the Director-General of the Army Medical Department, Victoria Street, S.W.; in the latter to the Director-General, Naval Medical Department, Northumberland Avenue, S.W., for forms of application.

(48) *Daily Nursing* (*Africa*).—A similar question was asked in "Everybody's Opinion" for March 28th last. Read what was said on the subject in the same column for April 11th.

(49) *Consumption* (*Birkdale*).—Japanese paper is the best for the purpose, but cheap calico squares, to be burnt when used, are perhaps better than anything else as a substitute for pockethandkerchiefs. The pocket should have a loose lining with a separate division into which the used squares can be placed, away from the clean ones and the other contents, and burnt altogether. There are also little bottles sold by chemists for carrying in the pockets for the reception of sputum, made with special facilities for thorough cleansing.

(50) *Combination Furniture* (*Nurse*).—If you explain what you want any furniture shop should be able to carry out the fittings in question. Messrs. Atkinson, Westminster Bridge Road, go in specially for hospital furniture, and have had much to do with the fitting up of nurses' homes. Messrs. Debenham (Wigmore Street. Cavendish Square) and Messrs. Maple (Tottenham Court Road) do the same; and the fittings of the matron's room at the Poplar Hospital, which are on these lines, were put up by Messrs. Shoolbred, Tottenham Court Road.

(51) *Orphanages* (*A. S.*).—See the list of such institutions in "Burdett's Hospitals and Charities" (Scientific Press, 428, Strand, W.C.); apply for particulars to those which appear to suit the case in question.

(52) *Monthly Nursing* (*Nurse B.*).—You will find a list of lying-in hospitals in Burdett's "Hospitals and Charities" (Scientific Press, 428, Strand, W.C.). Apply to the matrons for particulars of training given.

### Reading to the Sick.

LONELINESS.

**Motto.**

"Out of weakness were made strong."

**Verses.**

When a much-loved friend is nigh,
And we sit silently,
That silence is not solitude.

Prayer is the holy gate
To the chamber of Thy state,
Which nearer and more near to Thee
Doth lead us—everlastingly. —*Isaac Williams.*

Love thy God, and love Him only,
And thy breast will ne'er be lonely,
In that one great Spirit meet
All things mighty, grave, and sweet.
Vainly strives the soul to mingle
With a being of our kind;
Vainly hearts with hearts are twined,
For the deepest still is single.
An impalpable resistance
Holds like natures at a distance.
Mortal! love that Holy One,
Or dwell for aye alone. —*Aubrey de Vere.*

Passing soon and little worth
Are the things that tempt on earth—
Heavenward lift thy soul's regard:
God Himself is thy reward! —*Clark.*

The easy path in the lowland hath little of grand or new,
But a toilsome ascent leads on to a wide and glorious view!
Peopled and warm is the valley, lonely and chill the height,
But the peak that is nearer the storm-cloud is nearer the stars of light. —*F. R. Havergal.*

**Reading.**

"No man is called to a life of self-denial for its own sake. It is in order to a compensation which is always real and always proportionate."—*Drummond.*

"What is it that you cannot bear? Is it pain? No! You scorn the idea of being so poor a soldier, as to be unable to bear this or that bout of pain. You have gone through as bad, or worse, before, and you can set your teeth and bear it. You feel that the world is full of pain, and that you can, and will, bravely bear your share, and enjoy the respites between, and your many pleasures—love of friends, books, the beauty of Nature, the very keenness of perception, intensified by your nervous sufferings, which, while it enhances your pain, enhances your pleasure also. You say with Hezekiah, 'I will walk in state through all my years, in spite of the bitterness of my soul.' No, it is not this or that bout of actual pain which you cannot bear, and which makes you dash your head against the wall of your life. It is the being cut off from the life of others, from that union in love and work which others enjoy. You are alone. You have love given to you, but you suspect it springs from pity, and you sometimes feel only irritated by it. People are always giving it you—you want to be free to give, free to pick and choose, and not to be merely chosen by those who find time to stay and pick up the wounded. And, in the deepest relationship of life you are alone; you never have

'The thousand sweet, still joys of such
As hand in hand face earthly life.'

A great part of life, the best part of life, is a sealed book to you; you are a Levite, with 'no part nor inheritance in the (earthly) land of Israel. But the Lord God is your inheritance.' Do not those other words, addressed to the Levites, go home to you, 'Seemeth it but a small thing unto you to be brought nearer unto the Lord your God'?

"For you are brought so near to Him, that we, who are prosperous, can only hold up our hands in silence, before one who is so chosen out to share Christ's own life."—*L. H. M. Soulsby.*

## Figures and Tables

**Fig. 11. f1:**
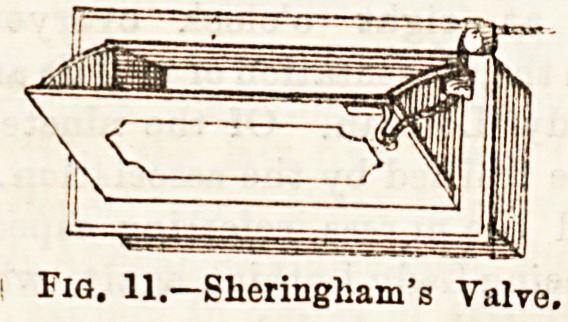


**Fig. 12. f2:**
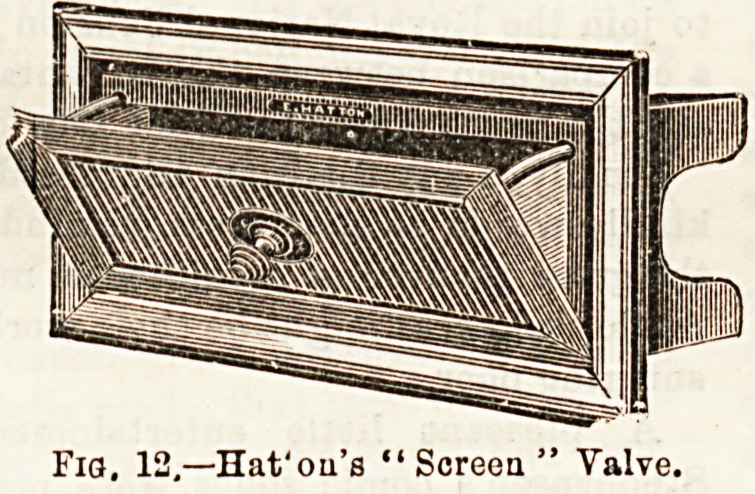


**Fig. 13. f3:**
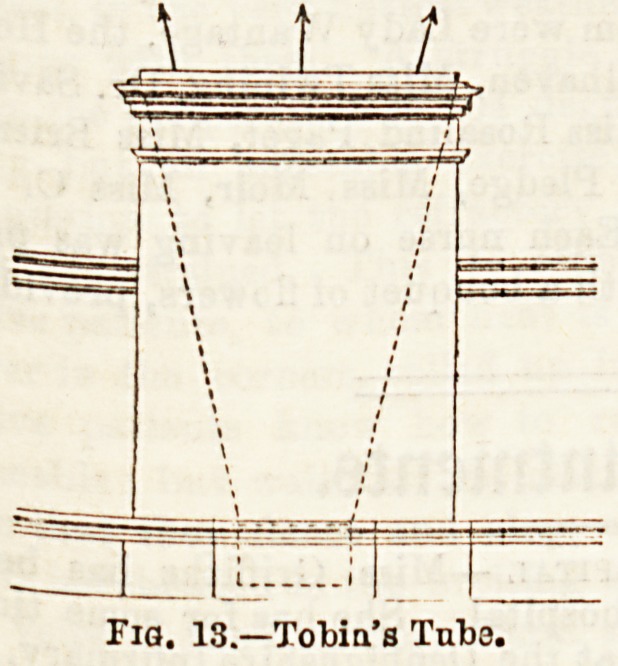


**Fig. 14. f4:**
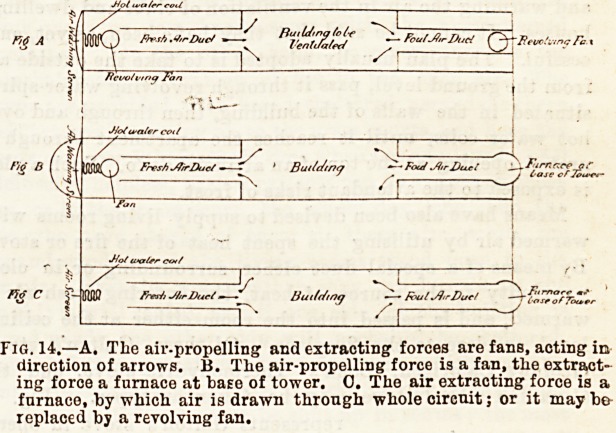


**Fig. 15. f5:**
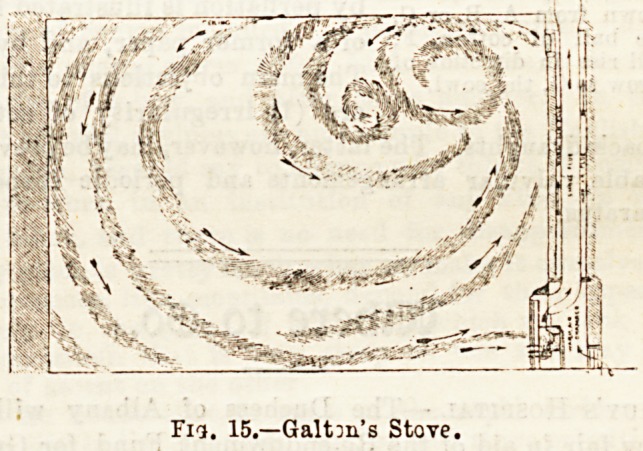


**Fig. 16 f6:**